# Concurrent positive skin tests to prophylactic antibiotics and rocuronium in two patients with life-threatening anaphylaxis after induction of anesthesia

**DOI:** 10.1186/s40981-021-00440-0

**Published:** 2021-04-20

**Authors:** Masako Yasuda, Katsuyuki Moriwaki, Yasuo M. Tsutsumi

**Affiliations:** 1 Department of Anesthesiology, Critical Care and Pain Medicine, National Hospital Organization Kure-Medical Center Chugoku Cancer Center, 3-1 Aoyamacho, Kure, Hiroshima, 737-0023 Japan; 2 Department of Anesthesiology, Hiroshima Hiramatsu Hospital, 11-27 Hijiyama Honmachi, Minami-ku, Hiroshima, 732-0816 Japan; 3 Department of Anesthesiology and Critical Care, Graduate School of Biomedical and Health Sciences, Hiroshima University, Kasumi 1-2-3 Minami-ku, Hiroshima, 734-8551 Japan; 4 Present address: Hiroshima, Japan

**Keywords:** Anaphylaxis, Anti-bacterial agents, Neuromuscular blocking agents, Adrenaline (epinephrine), Skin tests, General anesthesia

## Abstract

**Background:**

Prophylactic antibiotics and neuromuscular blocking agents (NMBA) are two of the major causative agents of anaphylaxis after induction of anesthesia.

**Case presentation:**

One female and one male patients (aged 29 and 69 years, respectively) had Ring and Messmer scale grade III anaphylaxis after administration of prophylactic antibiotics following induction of anesthesia. They showed typical hemodynamic and respiratory features of life-threatening anaphylaxis. Postoperative skin tests in these two patients were positive for antibiotics and concurrently positive for rocuronium.

**Conclusions:**

Our present report suggests the possibility that both prophylactic antibiotics and NMBA concurrently and synergistically enhance anaphylactic reaction and the necessity to differentiate an immune mechanism from non-immune mechanisms when anesthesiologists encounter concurrent positive skin tests for both antibiotics and NMBA.

## Highlights

- Antibiotics and NMBA showed concurrent positive skin test results in two patients.
- Both antibiotics and NMBA could synergistically enhance the signs of anaphylaxis.
- Non-immune mechanisms may be involved if skin tests are concurrently positive for NMBA.

## Background

Prophylactic antibiotics and neuromuscular blocking agent (NMBA) are two of the major causative agents of anaphylaxis during anesthesia [1–4]. Since prophylactic antibiotics should be administered intravenously during the interval beginning 60 min before incision to prevent surgical site infection [5], they are usually given before or immediately after the induction of anesthesia. At Kure Medical Center, life-threatening anaphylaxis of Ring and Messmer scale grade III occurred in this phase of anesthesia in three patients among 32,576 patients who underwent anesthesia from fiscal years 2007 to 2018. Among them, we report two patients who had concurrent positive results of skin tests for both antibiotics and NMBA. Written informed consent for the publication of this case report was obtained from the two patients.

## Case presentation

### Patients’ characteristics and the onset of the anaphylaxes

#### Case 1

A 29-year-old woman, ASA (PS) class 1, was scheduled to undergo uterine fibroid enucleation and right ovarian tumor enucleation under general anesthesia combined with epidural anesthesia. She had no history of allergies. After insertion of the epidural catheter, anesthesia was induced with fentanyl, remifentanil, propofol, and rocuronium, and the trachea was intubated. After administration of cefazolin 1 g before the start of surgery, blood pressure (BP) dropped to 50 mmHg or less, and did not respond to repeated bolus administration of ephedrine and phenylephrine, meanwhile heart rate (HR) increased rapidly from 50 to 90 beats/min, and arterial oxygen saturation of pulse oximetry (S_p_O_2_) decreased to 83%.

#### Case 2

A 69-year-old man, ASA (PS) class 3, was scheduled for retroperitoneoscopic prostatectomy for prostate cancer under combined general and epidural anesthesia. The patient had a history of rheumatoid arthritis, hypertension, hyperlipidemia, and endoscopic surgery for gastric cancer. He had a history of allergy to penicillin antibiotics. After placing an epidural catheter, anesthesia was induced using propofol and remifentanil. After administration of rocuronium, the trachea was intubated, and then cefotiam 1 g was administered. Before the start of surgery, the patient had persistent hypotension of systolic BP≤60 mmHg, which did not improve by repeated bolus administrations of ephedrine and phenylephrine. At the same time, HR rapidly increased from 40 to 70 beats/min. At the timing of starting surgery and feeding the retroperitoneal cavity with CO_2_ gas, S_p_O_2_ decreased to 92% at F_I_O_2_ 0.4.

### Cardiovascular and respiratory changes

Both patients had a HR increase accompanied by a progressive BP decrease. They also showed declines in both S_p_O_2_ at F_I_0_2_ 0.4 and EtCO_2_ under constant minute ventilation (MV). In case 1, EtCO_2_ decreased from 40 to 22 mmHg in association with an abrupt increase in the peak inspiratory pressure (PIP). Adrenaline (epinephrine) 50 μg, 75 μg were initially administered intravenously, in cases 1 and 2, respectively, and continuously given at 2 to 10 μg/min in both patients. Intravenous adrenaline immediately improved the refractory hypotension and increased EtCO_2_. In case 1, intravenous adrenaline also promptly reduced PIP. During the operating room stay, patients were infused 1750 and 1650 mL of crystalloid, respectively. Methylprednisolone 500 mg and chlorpheniramine 5 mg were also administered intravenously for both patients. Sugammadex was not used to treat anaphylaxis.

### Cutaneous signs

None of the two patients had cutaneous signs at the onset of anaphylaxis. Facial flushing became apparent after the restoration of BP with adrenaline administration in both patients.

### Serum tryptase and plasma histamine concentrations

Arterial blood was collected from the arterial lines to measure histamine and tryptase 30 to 40 min after the onset of anaphylaxis. The plasma histamine level was 23 ng/mL (normal range, 0.15 to 1.23 ng/mL) in case 1, and 1.34 ng/mL in case 2. In case 2, the total tryptase concentration was 3.4 μg/L (normal range, 1.2 to 5.7 μg/L).

### Outcomes

Surgical operations were stopped and postponed in both cases 1 and 2. Patients were treated in the intensive care unit and discharged uneventfully.

### Skin tests and reoperation

Patients were referred to dermatologists and underwent skin tests. Skin tests were performed 30 days after anaphylaxis for case 1, and 61 days for case 2. In cases 1 and 2, the antibiotics (cefazolin and cefotiam, respectively) and rocuronium had positive reactions in the intradermal test, although prick tests showed negative reactions. All other anesthetic agents used in each patient showed negative skin test reactivity. Reoperations were performed uneventfully on the 128th day after the anaphylaxis in case 1, and on the 181st day in case 2, while avoiding skin test-positive antibiotics and rocuronium.

## Discussion

### Clinical features and diagnosis

Our patients showed severe hypotension refractory to a repetitive dose of ephedrine and phenylephrine and concomitant HR increase. As for respiratory changes, our patients showed a decrease in both SpO_2_ and EtCO_2_. Generalized erythema or urticaria was not apparent, and facial flushing became apparent after the restoration of BP. Administration of adrenaline was effective for hemodynamic restoration in both cases and relief of bronchospasm in case 1. All these hemodynamic, respiratory, cutaneous features, and responses to adrenaline were compatible with those previous reports in patients with grade III anaphylaxis [1, 2, 6].

It is recommended that serum tryptase measurements be performed at 1 h as the first sample, 2 to 4 h as the second, and at least 24 h post-reaction onset as a baseline sample [2]. We measured only histamine concentration at the time of arterial line insertion in case 1. The significant elevation of histamine concentration in this case seemingly indicates that histamine was released from mast cells and basophils due to an anaphylactic reaction [7, 8]. However, elevated histamine and tryptase levels were not significant in case 2; thus, diagnosis for anaphylaxis was based on clinical signs and skin tests.

### Concurrent positive skin tests for both an antibiotic and an NMBA

A notable finding in this case report was that patients showed simultaneous positive results for antibiotics and rocuronium in skin tests. One study reported that patients with a positive history of antibiotic hypersensitivity had a higher incidence of positive skin tests for NMBAs [9]. Although the underlying mechanism is unknown, the concurrent positive skin tests for both an antibiotic and an NMBA may suggest that both agents had acted synergistically and exacerbated the severity of the anaphylaxis.

Skin tests alone cannot distinguish whether anaphylaxis is due to an immune or non-immune mechanism [10]. Specific IgE antibodies have been detected in some patients for both antibiotics and NMBA [11, 12]. Thus, anaphylaxis in our patients may be IgE antibody-mediated type I allergic reactions to both antibiotics and NMBA. However, it has been shown that NMBA may activate mast cells independently from IgE antibodies via the newly identified human Mas-related G-protein-coupled receptor member X2 (MRGPRX2) [10]. In our patients, we made no further investigation to identify whether the skin reaction was positive for IgE-mediated type I allergy or MRGPRX receptor-mediated response. The precise mechanisms, if determined, may provide information on the more rational use of agents for the anesthesia in reoperation.

## Conclusion

Our present report suggests the possibility that both prophylactic antibiotics and NMBA concurrently and synergistically enhance anaphylactic reaction and the necessity to differentiate an immune mechanism from non-immune mechanisms when anesthesiologists encounter concurrent positive skin tests for both antibiotics and NMBA.

## Data Availability

Not applicable
